# Long Non-coding RNA Uc.48+ Small Interfering RNA Alleviates Neuroinflammatory Hyperalgesia in Gp120-Treated Rats via the P2Y12 Receptor

**DOI:** 10.3389/fnins.2021.663962

**Published:** 2021-07-13

**Authors:** Lichao Peng, Bing Wu, Liran Shi, Lifang Zou, Lin Li, Runan Yang, Xiumei Xu, Guilin Li, Shuangmei Liu, Chunping Zhang, Shangdong Liang

**Affiliations:** ^1^School of Life Sciences, Xiamen University, Xiamen, China; ^2^Neuropharmacology Laboratory of Physiology Department, Medical School of Nanchang University, Nanchang, China; ^3^Department of Cell Biology, Medical School of Nanchang University, Nanchang, China; ^4^Jiangxi Provincial Key Laboratory of Autonomic Nervous Function and Disease, Nanchang, China

**Keywords:** dorsal root ganglia, HIV gp120-associated neuroinflammatory pain, long non-coding RNA, small interfering RNA, P2Y12 receptor

## Abstract

Human immunodeficiency virus envelope glycoprotein 120 (gp120) leads to hyperalgesia. Long non-coding RNAs are characterized by the lack of a protein-coding sequence and may contribute to the development and maintenance of inflammatory and neuroinflammatory pain. Rats with neuroinflammatory pain were established by gp120 treatment, which is featured by intensified pain behaviors. Long non-coding RNA uc.48+ was increased in the dorsal root ganglia of gp120-treated rats, and small interfering RNA that targets uc.48+ markedly alleviated hyperalgesia in gp120-treated rats. Notably, uc.48+ overexpression increased P2Y12 expression in control rats dorsal root ganglia and induced hyperalgesia. Uc.48+ small interfering RNA inhibited P2Y12 expression in gp120-treated rats. Uc.48+ potentiated P2Y12 receptor functions in the neurons and heterologous cells. Therefore, uc.48+ siRNA treatment reduced the upregulation of P2Y12 expression and function in DRG neurons, and, hence, alleviated hyperalgesia in gp120-treated rats.

## Introduction

Neuropathic and inflammatory pain due to human immunodeficiency virus (HIV) is a major symptom influencing almost 30% of infected patients, causing considerable societal and health burdens (Simpson et al., 2006; Hao, 2013; Schutz and Robinson-Papp, 2013). By activating macrophages, HIV-1 envelope glycoprotein 120 (gp120) causes the release of interleukin-1β and tumor necrosis factor-alpha (Jones et al., 2005). In “pain-positive” HIV-1 patients, the expression level of HIV-1 gp120 is markedly augmented (Wallace et al., 2007; Hoke et al., 2009; Yuan et al., 2014). The dorsal root ganglia (DRG) contain pseudounipolar neurons and primary afferent fibers that transmit noxious stimuli from the periphery to the central nervous system (Basbaum et al., 2009; Shi et al., 2018). Evidence suggests that gp120 can injure the primary sensory neurons (Meucci et al., 1998; Keswani et al., 2003; Chi et al., 2011; Mangus et al., 2014). *In vivo* studies have found that gp120 application into the rat sciatic nerves will produce hyperalgesia, and patients with HIV infection will rise inflammatory processes (Katayama et al., 2005; Kamerman et al., 2012; Hao, 2013; Yuan et al., 2014). Pro-inflammatory factors will cause the primary afferents and produce an increase in pain sensitivity (Basbaum et al., 2009; Kamerman et al., 2012; Hao, 2013).

Non-coding RNAs are characterized by the lack of a protein-coding sequence (Katayama et al., 2005; Patrushev and Kovalenko, 2014). Long non-coding RNAs (lncRNAs) contain over 200 nucleotides (Costa, 2010). LncRNAs can interact with DNAs, RNAs, or proteins and participate in regulating gene expression and protein functions (Rinn et al., 2007; Huarte et al., 2010; Cesana et al., 2011). LncRNAs expression changes are involved in the occurrence and development of neurological diseases (Qureshi et al., 2010; Pastori and Wahlestedt, 2012). Previous research showed that lncRNA uc.48+ is related to trigeminal neuralgia and diabetic neuroinflammatory pain. Uc.48+ contributed to diabetic neuropathic pain by upregulating the expression of P2X3 receptor in DRG or the release of calcitonin gene-related peptide in the spinal cords (Wang et al., 2016; Xiong et al., 2017). The activation of P2X and P2Y receptors is related to sensory neurotransmission (Burnstock, 2013, 2017). Reports have shown that the P2Y12 is an adenosine-5′-diphosphate (ADP)-responsive receptor expressed in the neurons and satellite glial cells of primary sensory ganglia, playing roles in developing inflammatory and neuroinflammatory pain (Kobayashi et al., 2006, 2008, 2013; Katagiri et al., 2012; Kawaguchi et al., 2015). Because allodynia and hyperalgesia are accompanied by peripheral sensitization originating from primary afferent neurons, the P2Y12 receptor in primary sensory neurons may participate in neuroinflammatory pain signaling in primary afferent transmission. Purinergic receptors play a role in HIV entry into macrophages (Matuszczak et al., 2017). Because analgesics for the treatment of HIV-associated neuroinflammatory pain are unavailable, we explore the role of lncRNA uc.48+ in P2Y12 receptor-mediated HIV-associated neuroinflammatory pain, which may formulate a new therapeutic approach for alleviating HIV-associated neuroinflammatory pain.

## Materials and Methods

### Animals

**FIGURE 1:**
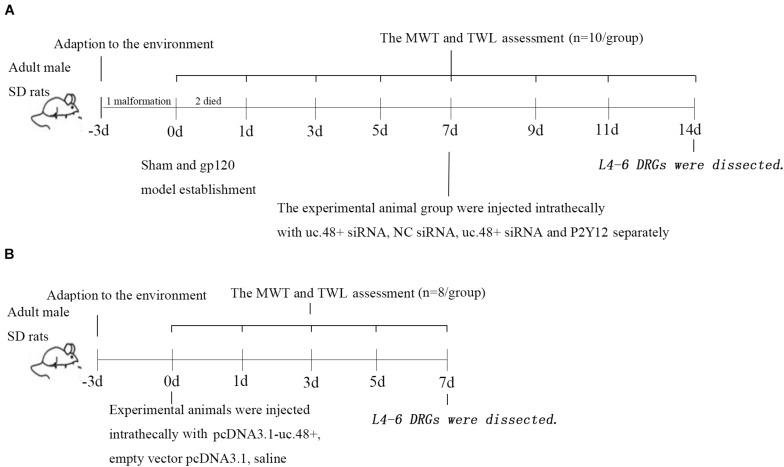
Timeline of study design. Timeline of gp120 animal model **(A)** and uc.48+ overexpression animal model **(B)**.

Male Sprague-Dawley rats (weighing 220 ± 10 g, approximately 7 weeks, Cat#: 70508, RRID: RGD_70508) were carried from the Experimental Animal Science Center of Nanchang University [approval number from Animal Welfare Committee, SYXK (Gan) 2015-0001], and the original source of rats come from Shanghai Animal Center. The experimental procedures were approved by the Animal Care and Use Committee of Nanchang University. All procedures were studied in accordance with the International Pain Research Association. The rats were placed in polypropylene plastic squirrel cages (four rats per cage) and kept with a quiet and well-ventilated system. The photoperiod was 12:12 h. The indoor temperature was maintained at 22–24°C, the humidity was maintained at approximately 50%, and water and food were replenished in time. The study was not preregistered. The timeline diagram and the number of animals required for each experiment are shown in Figures 1A,B.

### Neuroinflammatory Pain Model of Perineural gp120 Administration, *in vivo* Transfection of Small Interfering RNA or Plasmid

By the simple randomization method, the animals were randomly grouped as follows (10 animals per group): sham operation group (sham group), gp120 group (gp120 group), gp120-treated rats with lncRNA uc.48+ small interfering RNAs (siRNAs) group (gp120+ uc.48+ siRNA group), gp120-treated rats with scrambled siRNA group (gp120+ NC siRNA group), and gp120-treated rats with uc.48+ siRNA and the pcDNA3.1-P2Y12 plasmid group (gp120+ uc.48+ siRNA+ P2Y12 group). The procedures for the perineural administration of HIV-gp120 were the same as before (Wallace et al., 2007; Blackbeard et al., 2012). Briefly, we exposed the rats’ left sciatic nerve without perineurium damage under anesthesia with isoflurane (isoflurane can make animals wake up quickly and is used for short-term anesthesia). Oxidized regenerated cellulose strips (Johnson & Johnson, United States, Cat# 2081, 2017) were immersed in 250 μl salt solution containing 0.1% 400 ng gp120 (gp120, Sigma, United States, Cat#: SAE0071, 2017) and rat serum albumin for loosely wrapping the sciatic nerve. The sham group was treated with saline. According to the exclusion criteria, one deformed rat before the experiment was removed, and two rats died during the operation.

The authors followed the instructions of Entranster-*in vivo* for RNA transfection reagent (Engreen, China, Cat# 18668-11-1, 2017). A total of 5 μg siRNA (uc.48+ siRNA or scramble siRNA, Novobio Scientific Inc., Minhang, China) was intrathecally injected (under anesthesia with isoflurane) into the rats in the gp120+ uc.48+ siRNA, gp120+ scramble siRNA, and gp120 + uc.48 + siRNA + P2Y12 groups on day 7 after gp120 administration. In addition, 5 μg DNA (pcDNA3.1-P2Y12, Novobio Scientific Inc., Minhang, China) was simultaneously intrathecally injected (under anesthesia with isoflurane) into the rats in the gp120+ uc.48+ siRNA+ P2Y12 group on day 7 according to the DNA transfection instructions (Engreen, Beijing, China, Cat#18668-11-2, 2017).

SiRNA specific for uc.48+ and a negative control scrambled siRNA were provided by Invitrogen (United States). The sequences were as follows: uc.48+ siRNA target sequence 5′-GGCACTACTACTTGCAGAA-3′ and scramble siRNA target sequence 5′-UUCUCCGAACGUGUCACGUTT-3′. The same investigator measured the rats’ behavior on days 0, 1, 3, 5, 7, 9, 11, and 14 in the morning after the start of the experiment. On the 14th day, the rats were killed with carbon dioxide, and DRGs from L4-L6 on the left side were dissected (Basbaum et al., 2009; Shi et al., 2018).

### Overexpression of pcDNA3.1-uc.48+ Plasmid in the Control Rats

By the simple randomization method, rats were randomly grouped as follows (eight animals per group): control group (Ctr group), control animals treated with pcDNA3.1-uc.48+ (Ctr+ uc.48+ group), and control animals treated with the empty vector pcDNA3.1 (Ctr+ vector group). PcDNA3.1-uc.48+ or pcDNA3.1 was injected intrathecally into the Ctr+ uc.48+ and Ctr+ vector groups on day 0 using Entranster *in vivo* DNA transfection reagent. The control rats received the same volume of saline. The same investigator blind to the group assignments measured the rats’ behavior on days 0, 1, 3, 5, and 7 in the morning after the start of the experiments. On day 7, the rats were killed, and the same part DRGs were dissected.

### Assessment of Mechanical Withdrawal Threshold

The electronic, mechanical stimulator (Stoelting, Wood Dale, IL, United States) was used to measure the mechanical withdrawal threshold (MWT) in the rats. The maximum pressure value of the device is 50 g (Jia et al., 2017; Ge et al., 2019); the test needle was used to touch the left hind paw until the rats lifted their paw. A computer automatically recorded the value. Each rat was subjected to the test five times, and two stimuli were separated by an interval of 5 min and defined the average of the measured values as MWT.

### Assessment of Thermal Withdrawal Latency

The system of Thermal Paw Stimulation (BME-410C, Tianjin) was adopted to determinate the thermal withdrawal latency (TWL) in the rats. After being quiet, the plantar surface of the rat paw was exposed to radiant heat (Xie et al., 2017). The beam was turned off immediately once withdrawal responses appeared in the left hind paws. The paw withdrawal latency was the time shown on the screen. The time interval for measuring the hind paw was 5 min. The duration of the thermal stimulus was 30 s.

### Real-Time Polymerase Chain Reaction

Sample total RNA extraction was performed as described before (Basbaum et al., 2009; Shi et al., 2018). The sequences of primers were as follows: uc.48+, sense 5′-GCAAACTGGATGAGGAT-3′, antisense 5′-GTAGTGCCACAAGGAGA-3′; P2Y12, sense 5′-CTTCGTTCCCTTCCACTTTG-3′, antisense 5′-AGGGTGC TCTCCTTCACGTA-3′; β-actin, sense 5′-TAAAGACCTCTA TGCCAACACAGT-3, antisense 5′-CACGATGGAGGGG CCGGACTCATC-3′. Quantitative polymerase chain reaction (PCR) was executed using SYBR Green Master Mix. The expression levels of the target gene were calculated by a 2^–Δ^ ^Δ^ ^*CT*^ method.

### Western Blotting

The ganglia or cells were homogenized with radio immunoprecipitation assay lysis (Applygen, Beijing, China, Cat# C1053+, 2017). The concentration of protein supernatant was measured by the Lowry method, then diluted with protein loading buffer (Transgen Biotech, China) and bathed in boiling water for 5 min. Aliquots of protein were separated using 10% sodium dodecyl sulfate–polyacrylamide gel electrophoresis, followed by transfer to polyvinylidene fluoride membrane (Millipore, United States). The membrane was blocked for 2 h, then incubated with rabbit anti-P2Y12 (1:2,000 dilutions, Alomone Labs, Israel), rabbit anti-p38 mitogen-activated protein kinase (p38 MAPK), rabbit anti-phosphorylated-p38 (P-p38 MAPK) (1:1,000 dilutions, Cell Signaling Technology, United States), and mouse monoclonal anti-β-actin (1:800 dilutions, ZSGB-Bio) antibodies at 4°C overnight. After being washed, the membrane was incubated with the secondary antibody (anti-mouse IgG, goat anti-rabbit IgG, 1:2,000, ZSGB-Bio). The Bio-Rad system was used for labeled proteins visualized. Image-J was applied to quantify the results (Zhao et al., 2019).

### *In situ* Hybridization

*in situ* hybridization (ISH) was used to identify uc.48+ RNA expression in the ganglia. The ganglia were prepared as previously described and sectioned at a 15 μm thickness. All of the ISH solutions and appliances applied diethylpyrocarbonate. The slides were incubated with proteinase K (Boster, Wuhan, China) (37°C, 5 min) after deparaffinization and hydration. Then, the tissues were incubated with pre-hybridization solution (Boster, Wuhan, China) at 42°C for 4 h, followed by incubation with sense or antisense probes at 46°C for 18 h. Then, an anti-digoxigenin alkaline phosphatase that was conjugated to an antibody was incubated for 1 h, followed by washing with maleic acid buffer with Tween. Before treatment with nitro blue tetrazolium/5-bromo-4-chloro-3-indolyl phosphate developing solution (Roche, Basel) at 37°C without light, the sections were washed using a pre-staining buffer. Phosphate-buffered saline was used to terminate the reaction at 4°C for 16 h. Finally, 0.5% Bismarck Brown Y was used to counterstain the slides. The tissues were mounted after dehydration.

### Co-transfection of HEK 293 Cells With P2Y12 Plasmid and pcDNA3.1-uc.48+ Plasmid or uc.48 Small Interfering RNA

HEK 293 cells (American Type Culture Collection, maximum number of passages for cell lines was 13) were cultivated in Dulbecco’s modified Eagle medium (Gibco, United States) supplemented with 10% fetal bovine serum (Biological Industries, Cromwell, CT, United States) and 1% penicillin–streptomycin solution (Solarbio, Beijing, China) in an incubator at 37°C with 5% carbon dioxide. The P2Y12 and uc.48+ plasmids were co-transfected by FuGENE 6 (Promega, United States, Cat#: E2691, 2018). The P2Y12 plasmid and uc.48+ siRNA were co-transfected using jetPRIME (Polyplus Transfection, France). Twenty-four hours after transfection, cells were collected for Western blot or cyclic adenosine monophosphate (cAMP) measurements.

### Cyclic Adenosine Monophosphate Assay

The determination of intracellular cAMP concentrations was modified from the previously described protocol (Zhang et al., 2017; Zou et al., 2019). After 24 h of transfection, all the wells were added with 10 μM ADP (Sigma, United States) and incubated at 37°C for 5 min (Zhu et al., 2015). The mixture was collected in 100 μl phosphate-buffered saline, frozen at −80°C for 45 min, and then thawed in a 37°C water bath three times. After being centrifuged at 1,500 × *g* for 10 min, the supernatant was collected. Also, the cAMP Elisa Kit (Elabscience Biotechnology, China) was used to quantify cAMP concentrations.

### Recording Voltage-Gated Ca^2+^ Currents

Primary cultures of DRG neurons from neonatal Sprague-Dawley rats were established previously (Lechner et al., 2004; Sun et al., 2019). DRGs from rat pups were removed, and the attached nerves were cut. Then, the ganglia were incubated with 1.5 mg/ml collagenase and 3.0 mg/ml dispase II (Sigma, United States) at 37°C for 20 min, followed by 0.25% trypsin treatment at 37°C for 15 min. The ganglia were dissociated by trituration, resuspended in Dulbecco’s modified Eagle Medium, 5% fetal bovine serum, 1% penicillin and streptomycin, 10 μg/ml insulin, and 50 ng/ml nerve growth factor (Sigma, United States), seeded in 35 mm dishes, and incubated for 3–8 days.

Uc.48+ overexpression was achieved *via* uc.48+ plasmid attached with green fluorescent protein (2.5 μg) using FuGENE 6 transfection reagents. The neurons were incubated with 200 pmol/L gp120. In addition, uc.48+ siRNA (1.87 μg) was transfected with jetPRIME. The dishes were treated for 24 h.

The patch electrodes were filled with an internal solution containing the following (in millimolar): 130 cesium chloride, 20 tetraethylammonium chloride, 0.24 calcium chloride, 10 4-(2-hydroxyethyl)-1-piperazineethanesulfonic acid, 10 glucose, 5 ethylene glycol-bis(β-aminoethyl ether)-N,N,N′,N′-tetraacetic acid, 2 guanosine 5′-triphosphate lithium salt, and 2 adenosine 5′-triphosphate magnesium salt; the pH was adjusted to 7.4 with cesium chloride. The external solution consisted of (in millimolar) 120 sodium chloride, 20 tetraethylammonium chloride, 3 potassium chloride, 2 magnesium chloride, 5 calcium chloride, 20 glucose, and 10 4-(2-hydroxyethyl)-1-piperazineethanesulfonic acid, and the pH was adjusted to 7.3 with sodium hydroxide. Whole-cell I_*Ca*_ was elicited by 30 ms depolarizations from a holding potential of −80 to 0 mV at a frequency of 4/min. An online leak subtraction protocol was used to correct the leakage currents.

### Statistical Analysis

Statistical analysis was performed by means ± standard error of the mean (SEM). The experimental data were analyzed by SPSS 20 and graphed by GraphPad Prism 5 software. Unpaired *t*-test and one-way analysis of variance were used to determine statistical significance for comparison between two groups and multiple comparisons, respectively. A *p*< 0.05 was statistically significant.

## Results

### Uc.48+ Was Increased in Human Immunodeficiency Virus gp120-Induced Neuroinflammatory Pain Rats

**FIGURE 2:**
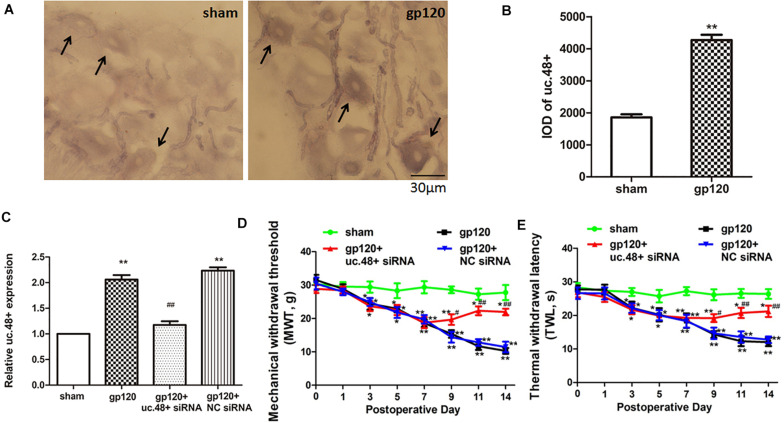
LncRNA uc.48+ was involved in gp120-induced neuroinflammatory pain. **(A)** Uc.48+ was expressed in cytoplasm of DRG neurons (scale bar: 30 μm). **(B)** ISH showed uc.48+ expression in gp120 rats was markedly higher. **(C)** Real-time PCR results showed that uc.48+ siRNA downregulated expression of uc.48+. **(D,E)** After treatment with siRNA targeting uc.48+, MWT **(D)** and TWL **(E)** in gp120-treated rats were alleviated. *n* = 10 rats per group. Data are displayed as means ± SEM. ^∗^*p* < 0.05, ^∗∗^*p* < 0.01 vs. sham group, ^#^*p* < 0.05, ^##^*p* < 0.01 vs. gp120 group.

Uc.48+ was localized to the cytoplasm of the DRG neurons, and the ISH images indicated the uc.48+ integral optical density in gp120 rats that were markedly higher than sham rats (Figures 2A,B). The real-time PCR analysis showed that uc.48+ levels in gp120 rats were higher than sham rats (*p* < 0.01; Figure 2C). Our data suggest that the upregulation of uc.48+ expression may be involved in the pathological process of HIV gp120-induced neuroinflammatory pain.

### Uc.48+ Promoted Mechanical and Thermal Hyperalgesia in the gp120-Treated Rats

The mechanical and thermal hyperalgesia were assessed from day 1 to 14 after the gp120 treatment. Both MWT and TWL of gp120 rats were significantly decreased compared with sham rats from day 3 to 14 (*p* < 0.05, Figures 2D,E). These suggest that uc.48+ may have promoted mechanical and thermal hyperalgesia in gp120-treated rats.

Next, we investigated whether the silencing of uc.48+ expression in gp120-treated rats by siRNA could attenuate HIV gp120-induced neuroinflammatory pain. Seven days after gp120 treatment, the animals were transfected with uc.48+ siRNA. After transfection with uc.48+ siRNA, the uc.48+ expression in gp120 rats was reduced (*p* < 0.01; Figure 2C), and the MWT was markedly increased (*p* < 0.05, Figure 2D). The TWL in the gp120 + uc.48+ siRNA group shows the same trend (Figure 2E). No differences were found in these parameters between gp120 + NC siRNA and gp120 groups (*p* > 0.05). The data reveal that the knockdown of uc.48+ alleviated hyperalgesia in rats with gp120 treatment.

**FIGURE 3:**
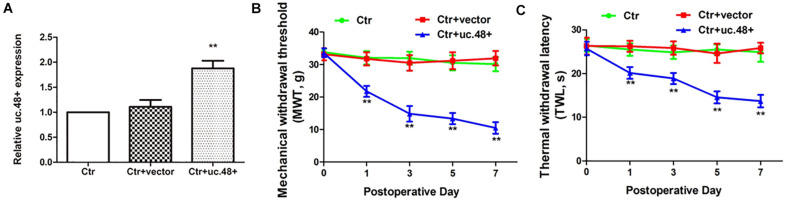
Overexpression of uc.48+ by intrathecal injection induced pain behaviors in control rats. **(A)** Real-time PCR analysis showed that rats treated with uc.48+ had higher expression of uc.48+ compared with control group. Overexpression of uc.48+ lowered MWT **(B)** and TWL **(C)**. *n* = 8 rats per group. Data are displayed as means ± SEM. ^∗∗^*p* < 0.01 vs. control group.

Given the upregulation of uc.48+ expression in response to the HIV gp120 treatment, we investigated whether the overexpression of uc.48+ could induce neuroinflammatory pain similar to that induced by the gp120 treatment in the control rats. Full-length uc.48+ plasmid was applied *via* intrathecal injection to the control rats. After transfection with uc.48+, uc.48+ expression was increased (*p* < 0.01; Figure 3A). Meanwhile, the MWT and TWL were measured to determine whether the uc.48+ overexpression in control rats induced nociceptive behavior. The MWT and TWL in the ctr + uc.48+ rats were markedly decreased compared with the control or ctr + vector rats from day 1 to 7 after transfection (*p* < 0.01; Figures 3B,C). The data manifest that the overexpression of uc.48+ produced pain behaviors in the control rats similar to those observed after the gp120 treatment.

### Uc.48+ Small Interfering RNA Treatment Alleviated Hyperalgesia by Downregulating P2Y12 Receptor

**FIGURE 4:**
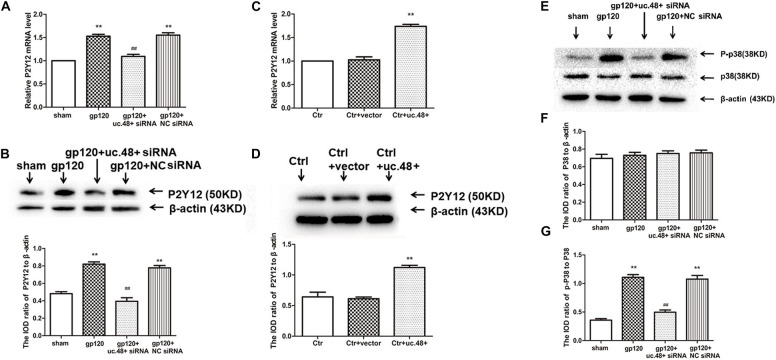
Effects of uc.48+ on P2Y12 receptor expression and activation of P2Y12 downstream P38 MAPK pathway *in vivo*. Real-time PCR **(A)** and Western blotting **(B)** analyses showed siRNA silencing of uc.48+ downregulated P2Y12 receptor expression. *n* = 10 rats per group. Data are displayed as means ± SEM. ^∗∗^*p* < 0.01 vs. sham group,^ ##^*p* < 0.01 vs. gp120 group. Real-time PCR **(C)** and Western blotting **(D)** results showed that overexpression of uc.48+ upregulated P2Y12 receptor levels in control rat DRG. *n* = 8 rats per group. Data are displayed as means ± SEM. ^∗∗^*p* < 0.01 vs. control group. **(E–G)** Uc.48+ siRNA lowered upregulated p-P38 MAPK levels in gp120 group. *n* = 10 rats per group. Data are displayed as means ± SEM. ^∗∗^*p* < 0.01 vs. sham group, ^##^*p* < 0.01 vs. gp120 group.

P2Y12 receptor is related to neuroinflammatory pain; both P2Y12 messenger RNA (mRNA) and protein were assayed to determine whether the P2Y12 receptor was a target for uc.48+. Gp120 treatment resulted in a significant increase in the mRNA and protein expression of P2Y12 (*p* < 0.01; Figures 4A,B). These results revealed a relationship between uc.48+ and P2Y12 receptor. We further found that following the uc.48+ siRNA application, the P2Y12 mRNA and protein expression were markedly decreased (*p* < 0.01; Figures 4A,B). No significant difference was found between gp120 + NC siRNA and gp120 groups (*p* > 0.05; Figures 4A,B). Our results demonstrate that the uc.48+ siRNA application inhibited upregulated P2Y12 receptors in gp120 rats.

**FIGURE 5:**
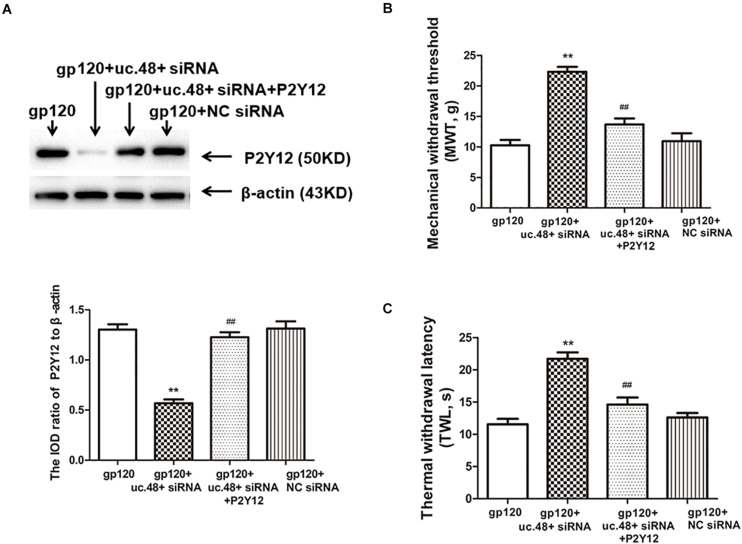
Overexpression of P2Y12 rescued effects of uc.48+ siRNA treatment on downregulated expression of P2Y12 receptor and hyperalgesia in gp120 group. **(A)** Western blotting results showed that downregulation of DRG P2Y12 protein in gp120-treated rats with uc.48+ siRNA was significantly rescued by overexpression of P2Y12. **(B,C)** Upregulation of MWT and TWL was rescued by overexpression of P2Y12. *n* = 10 rats per group. Data are displayed as means ± SEM. ^∗∗^*p* < 0.01 vs. gp120-treated rats, ^##^*p* < 0.01 vs. gp120-treated with uc.48+ siRNA group.

To further determine whether uc.48+ might target the P2Y12 receptor, the roles of the uc.48+ overexpression on the P2Y12 expression *in vivo* were assessed *via* real-time quantitative PCR and Western blotting in the control rats transfected with the pcDNA3.1-uc.48+. The data revealed P2Y12 mRNA and protein expression levels were remarkably enhanced after pcDNA3.1-uc.48+ transfection in these rats (*p* < 0.01; Figures 4C,D). No alteration of P2Y12 expression was found after transfection of empty vector (*p* > 0.05; Figures 4C,D). In addition, the P2Y12 overexpression increased the protein levels of P2Y12 after the uc.48 siRNA treatment (*p* < 0.01; Figure 5A). Meanwhile, the MWT and TWL were decreased after overexpression of P2Y12 in the gp120 plus uc.48+ siRNA group (*p*< 0.01; Figures 5B,C). Thus, P2Y12 overexpression could hinder the influences of uc.48+ siRNA treatment on the MWT and TWL in the gp120 rats. Consequently, uc.48+ may positively regulate the P2Y12 receptor to promote mechanical or thermal hyperalgesia.

P2Y12 receptor also mediates p38 MAPK activation, resulting in elevated pain sensitivity (Kobayashi et al., 2008). The phosphorylation of p38 MAPK is a marker of its activation (Zhao et al., 2019). To determine the status of p38 MAPK activation, the ratio of phospho-p38 MAPK to total p38 MAPK was analyzed. There was no conspicuous difference that exists in the ratio of p38 MAPK to β-actin between the gp120 and sham groups (*p* > 0.05; Figures 4E,F). In the gp120 group, the P-p38 MAPK expression level was higher than that of the sham group (Figures 4E,G). After treated with uc.48+ siRNA, the P-p38 MAPK levels were lower (*p* < 0.01; Figures 4E,G). These parameters had no conspicuous difference between the gp120 + NC siRNA and gp120 groups (*p* > 0.05; Figures 4E,G). Our results indicate that the application of uc.48+ siRNA decreased upregulated P2Y12 receptor, which was accompanied by the downregulated activation of p38 MAPK in gp120 rats.

### P2Y12 Receptor Played a Role in uc.48+ -Involved Pathological Process of gp120-Induced Neuroinflammatory Pain

**FIGURE 6:**
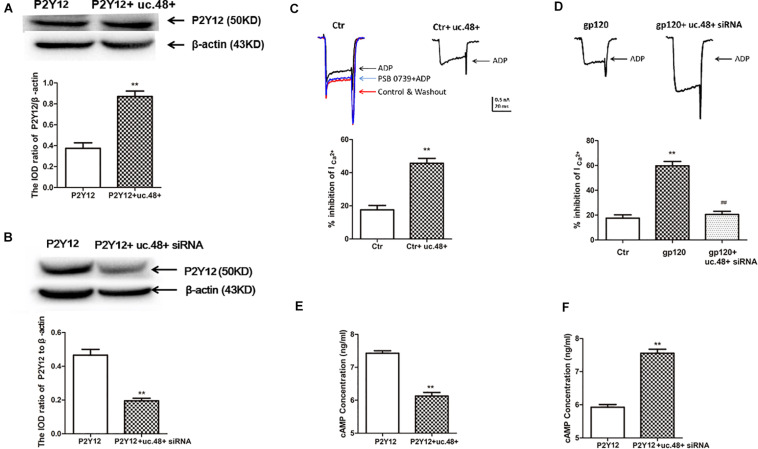
LncRNA uc.48+ positively regulated expression and activity of P2Y12 receptor. **(A)** P2Y12 protein content co-transfected with P2Y12 and uc.48+ plasmids was increased compared with transfected with P2Y12 plasmid alone in HEK293 cells. **(B)** P2Y12 protein content co-transfected with P2Y12 plasmid and uc.48+ siRNA was decreased in HEK293 cells. *n* = 6 independent cultures. Data are displayed as means ± SEM. ^∗∗^*p* < 0.01 vs. P2Y12 plasmid alone group. **(C,D)** Effects of uc.48+, gp120, and uc.48+ siRNA treatment on voltage-gated Ca^2+^ current mediated by P2Y12 receptor agonists in neurons. *n* = 12 independent cultures. Data are displayed as means ± SEM. ^∗∗^*p* < 0.01 vs. control group, ^##^*p* < 0.01 vs. gp120 treatment group. **(E,F)** ADP-induced intracellular cAMP levels were measured in HEK293 cells. *n* = 5 independent cultures. Data are displayed as means ± SEM. ^∗∗^*p* < 0.01 vs. P2Y12 group.

Western blotting results indicated the expression of P2Y12 protein in HEK293 cells co-transfected by uc.48+ and P2Y12, which was markedly higher than transfected only by P2Y12 plasmid (Figure 6A), whereas P2Y12 protein expression co-transfected with P2Y12 plasmid and uc.48+ siRNA was markedly lower than transfected only by P2Y12 plasmid (Figure 6B). ADP inhibits endogenous P2Y12 receptor-mediated Ca^2+^ current in rat neurons (Lechner et al., 2004). EGFP-uc.48+ plasmids were transiently expressed in the DRG neurons. Patch results revealed that the P2Y12 agonist ADP (100 μM) inhibited the voltage-gated Ca^2+^ currents in the rat DRG neurons and PSB 0739 (10 μM), the P2Y12 receptor antagonist, antagonized this inhibition (Figure 6C). ADP enhanced the inhibition treated with uc.48+ (Figure 6C). We further studied whether gp120 treatment had a direct effect on P2Y12 receptor agonist-mediated voltage-gated Ca^2+^ currents in neurons. The results showed that the gp120 treatment increased inhibition in the rat DRG neurons, and the upregulated inhibition induced by the gp120 treatment was reduced by uc.48+ siRNA treatment (Figure 6D). These results demonstrate that the overexpression of uc.48+ enhanced P2Y12 receptor activity in the rat DRG neurons.

P2Y12 receptor activation has been found to have a direct effect on adenylyl cyclase, causing its inhibition and reducing the production of cAMP (Unterberger et al., 2002). We examined whether the co-expression of uc.48+ and P2Y12 receptor in HEK293 cells could influence P2Y12-mediated effects on adenylyl cyclase. The intracellular cAMP concentrations induced by ADP in HEK293 cells were measured (Zou et al., 2019). The co-transfection of uc.48+ and P2Y12 plasmid caused a decrease in the cAMP levels in the HEK293 cells (Figure 6E). Consistent with the potentiation of P2Y12 receptor activity by uc.48+, the siRNA silencing of uc.48+ significantly elevated the intracellular cAMP levels (Figure 6F). Our data suggested that uc.48+ may have strengthened the expression and activity of the P2Y12 receptor in the heterologous cells.

## Discussion

Mechanical and thermal hypersensitivity occurs in rats with HIV gp120-associated inflammatory and neuroinflammatory pain (Wallace et al., 2007). The altered expression of lncRNAs is related to nervous system diseases (Qureshi et al., 2010; Pastori and Wahlestedt, 2012). The real-time quantitative PCR and ISH test in this study demonstrated that lncRNA uc.48+ was markedly elevated in the rats’ DRG with gp120-caused peripheral neuropathy. Thus, lncRNA uc.48+ may be a crucial regulator of gp120-induced neuroinflammatory pain, and targeting uc48+ could be an efficient therapy for clinical neuroinflammatory diseases. This study indicated that the increased uc.48+ in DRG of the gp120 rats had a relationship with heightened mechanical and thermal hyperalgesia. Moreover, siRNA silencing of uc.48+ reversed both gp120-induced mechanical and thermal hyperalgesia. The results indicated that upregulated uc.48+ in DRG might be a key factor in gp120-induced neuroinflammatory pain. Also, the overexpression of uc.48+ in the control rats DRG resulted in mechanical and thermal hyperalgesia, suggesting that uc.48+ is necessary and sufficient to induce neuroinflammatory pain behaviors in rats.

The activation of P2Y receptors is involved in the nociceptive transmission of inflammatory and neuroinflammatory pain (Kobayashi et al., 2008; Burnstock, 2009; Katagiri et al., 2012; Burnstock, 2013, 2017), whereas P2Y12 receptors play an important role in this scenario (Kobayashi et al., 2006; Katagiri et al., 2012; Burnstock, 2013; Kawaguchi et al., 2015). This study showed that P2Y12 mRNA and protein in the DRG of the gp120 rats were significantly increased, whereas treatment with uc.48+ siRNA could inhibit such upregulation of P2Y12 expression. This is associated with the result that siRNA silencing of uc.48+ mitigated gp120-induced mechanical and thermal hyperalgesia. P2Y12 receptor could be expressed in neurons in the primary sensory ganglia (Kawaguchi et al., 2015). Peripheral sensitization of inflammatory and neuroinflammatory pain originates from primary afferent neurons. P2Y12 receptors expressed in neurons might be involved in HIV gp120-induced neuroinflammatory pain.

The P2Y12 agonist ADP inhibits endogenous P2Y12 receptor-mediated Ca^2+^ currents in rat neurons (Lechner et al., 2004). Our data showed that ADP also inhibited voltage-gated Ca^2+^ currents in the rat DRG neurons, and this inhibition was antagonized by the P2Y12 receptor antagonist PSB 0739. After transfecting DRG neurons with uc.48+ plasmid, the inhibition was increased in the presence of ADP. Moreover, the gp120 treatment also enhanced the inhibition induced by ADP in the rat DRG neurons. After the siRNA silencing of the uc.48+ expression, the upregulated inhibition of voltage-gated Ca^2+^ currents due to gp120 treatment was weakened in the rat DRG neurons. P2Y12 receptor activation resulted in the inhibition of adenylyl cyclase and reduced the production of cAMP (Unterberger et al., 2002). After the co-transfection of uc.48+ and P2Y12 plasmid, the intracellular cAMP levels induced by ADP in HEK293 cells were significantly decreased. In contrast, uc.48+ siRNA treatment markedly elevated the intracellular cAMP concentrations. These observations suggest that overexpressed uc.48+ may have strengthened the expression and activity of the P2Y12 receptor in the rat DRG neurons and heterologous cells. Therefore, uc.48+ siRNA application reduced the upregulation of P2Y12 expression and activity in the DRG neurons in the gp120-treated rats. Works of literature have shown that lncRNAs interact with proteins, and uc.48+ may upregulate P2Y12 expression and activity by interaction. Uc.48+ has been found to be involved in other types of pain, but it has not been reported that uc.48+ mediates pain by regulating the P2Y12 receptor. Therefore, it is still necessary to further characterize whether this pathway is involved in other types of pain. Despite these limitations, our findings provide a rationale for clinical neuroinflammatory diseases.

## Conclusion

In summary, the upregulation of P2Y12 expression and activity in DRG neurons facilitated both mechanical and thermal hyperalgesia caused by gp120 application. Uc.48+ played a key role in the pathogenesis of neuropathic pain by regulating the P2Y12 receptor. The silencing of uc.48+ was able to reduce the upregulation of P2Y12 expression and activity in the DRG neurons in the gp120 rats. Consequently, the uc.48+ siRNA knockdown significantly alleviated HIV gp120-induced neuroinflammatory pain.

## Data Availability Statement

The raw data supporting the conclusions of this article will be made available by the authors, without undue reservation.

## Ethics Statement

The animal study was reviewed and approved by the Experimental Animal Science Center of Nanchang University. Written informed consent was obtained from the owners for the participation of their animals in this study.

## Author Contributions

LP, BW, and SDL conceived and designed the research. LP, BW, LS, LZ, LL, RY, XX, GL, SML, and CZ analyzed and interpreted the data. LP and BW performed the statistical analysis and drafted the manuscript. SDL revised the article and the study idea was from SDL. All authors contributed to the article and approved the submitted version.

## Conflict of Interest

The authors declare that the research was conducted in the absence of any commercial or financial relationships that could be construed as a potential conflict of interest.
