# Corrigendum: Long Non-coding RNA Uc.48+ Small Interfering RNA Alleviates Neuroinflammatory Hyperalgesia in Gp120-Treated Rats via the P2Y12 Receptor

**DOI:** 10.3389/fnins.2021.746945

**Published:** 2021-11-26

**Authors:** Lichao Peng, Bing Wu, Liran Shi, Lifang Zou, Lin Li, Runan Yang, Xiumei Xu, Guilin Li, Shuangmei Liu, Chunping Zhang, Shangdong Liang

**Affiliations:** ^1^School of Life Sciences, Xiamen University, Xiamen, China; ^2^Neuropharmacology Laboratory of Physiology Department, Medical School of Nanchang University, Nanchang, China; ^3^Department of Cell Biology, Medical School of Nanchang University, Nanchang, China; ^4^Jiangxi Provincial Key Laboratory of Autonomic Nervous Function and Disease, Nanchang, China

**Keywords:** dorsal root ganglia, HIV gp120-associated neuroinflammatory pain, long non-coding RNA, small interfering RNA, P2Y12 receptor

In the original article, there was a mistake in Figure 4 as published. Incorrect images were used in Figures 4D,E. The corrected Figures 4D,E appear below.

**Figure 4:**
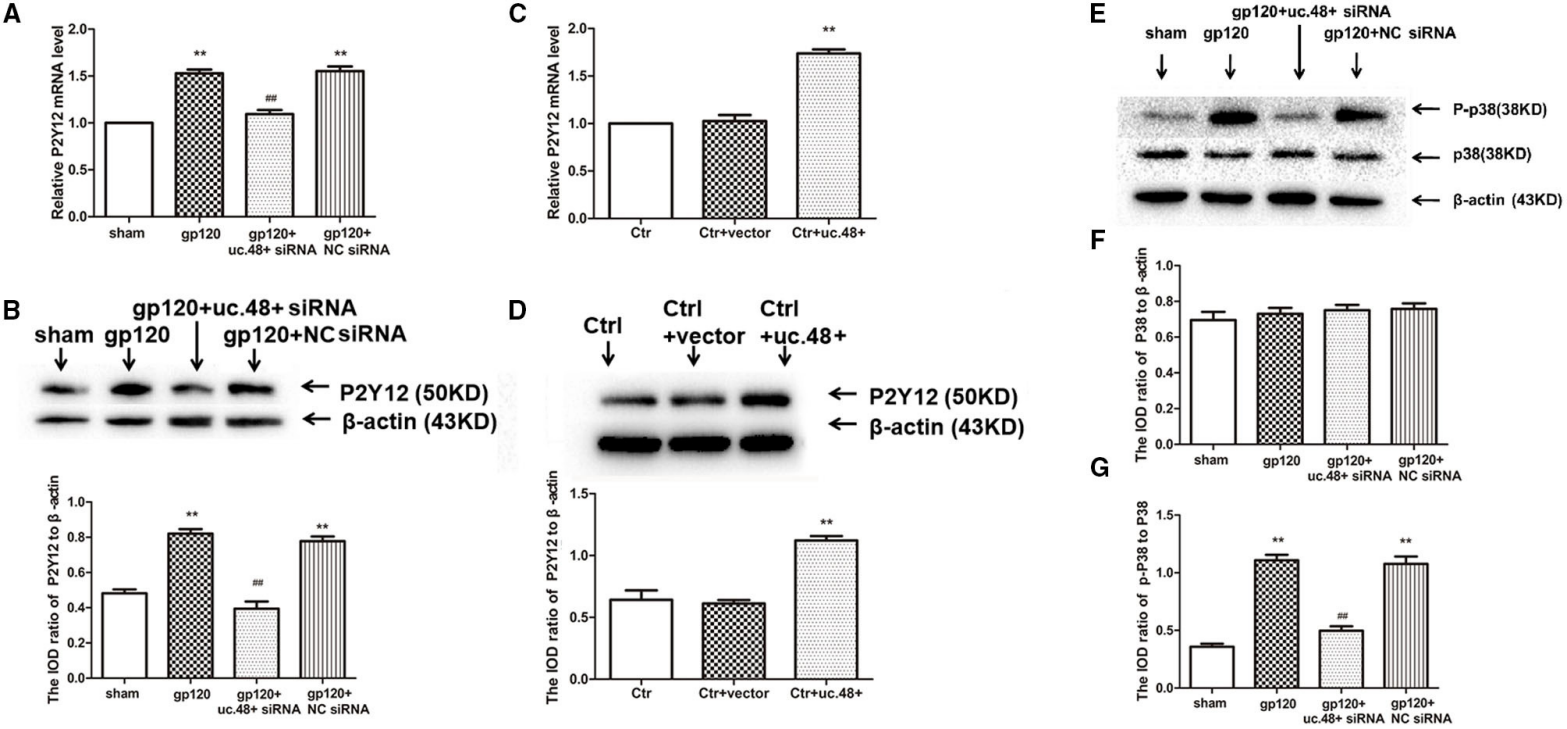
Effects of uc.48+ on P2Y12 receptor expression and activation of P2Y12 downstream P38 MAPK pathway *in vivo*. Real-time PCR **(A)** and Western blotting **(B)** analyses showed siRNA silencing of uc.48+ downregulated P2Y12 receptor expression. *n* = 10 rats per group. Data are displayed as means ± SEM. **p < 0.01 vs. sham group, ^##^*p* < 0.01 vs. gp120 group. Real-time PCR **(C)** and Western blotting **(D)** results showed that overexpression of uc.48+ upregulated P2Y12 receptor levels in control rat DRG. *n* = 8 rats per group. Data are displayed as means ± SEM. **p < 0.01 vs. control group. **(E–G)** Uc.48+ siRNA lowered upregulated p-P38 MAPK levels in gp120 group. *n* = 10 rats per group. Data are displayed as means ± SEM. **p < 0.01 vs. sham group, ^##^*p* < 0.01 vs. gp120 group.

The authors apologize for this error and state that this does not change the scientific conclusions of the article in any way. The original article has been updated.

## Publisher's Note

All claims expressed in this article are solely those of the authors and do not necessarily represent those of their affiliated organizations, or those of the publisher, the editors and the reviewers. Any product that may be evaluated in this article, or claim that may be made by its manufacturer, is not guaranteed or endorsed by the publisher.

